# The Role of Osteopontin in Tumor Progression Through Tumor-Associated Macrophages

**DOI:** 10.3389/fonc.2022.953283

**Published:** 2022-07-08

**Authors:** Yuying Tan, Lei Zhao, Yong-Guang Yang, Wentao Liu

**Affiliations:** ^1^ Key Laboratory of Organ Regeneration & Transplantation of the Ministry of Education, The First Hospital of Jilin University, Changchun, China; ^2^ National–Local Joint Engineering Laboratory of Animal Models for Human Diseases, Jilin University, Changchun, China; ^3^ International Center of Future Science, Jilin University, Changchun, China

**Keywords:** osteopontin, tumor associated macrophage, tumor microenvironment, tumor progression, immune regulation

## Abstract

Osteopontin (OPN) is a multifunctional phosphorylated protein. It is widely involved in solid tumor progression, such as intensification of macrophage recruitment, inhibition of T-cell activity, aggravation of tumor interstitial fibrosis, promotion of tumor metastasis, chemotherapy resistance, and angiogenesis. Most of these pathologies are affected by tumor-associated macrophages (TAMs), an important component of the tumor microenvironment (TME). TAMs have been extensively characterized, including their subsets, phenotypes, activation status, and functions, and are considered a promising therapeutic target for cancer treatment. This review focuses on the interaction between OPN and TAMs in mediating tumor progression. We discuss the strategies for targeting OPN and TAMs to treat cancer and factors that may affect the therapeutic outcomes of blocking OPN or depleting TAMs. We also discuss the role of cancer cell- vs. TAM-derived OPN in tumorigenesis, the mechanisms of how OPN affects TAM recruitment and polarization, and why OPN could mediate anti-tumor and pro-tumor effects, as well as previously reported discrepancies.

## Introduction

Osteopontin (OPN) is encoded by the secreted phosphoprotein 1 (SPP1) gene. OPN is named for its role as a bridge between cells and hydroxyapatite through the function of Arg-Gly-Asp (RGD) and polyaspartic acid motifs (1). It has also been shown that activated T cells express high levels of OPN in the early stage, which is therefore named the early T-lymphocyte activation-1 (Eta-1) protein in the view of immunology (2).

The expression of OPN is negatively correlated with the prognosis of patients with colorectal, head, and neck cancers (3, 4), and it has been identified as a biomarker for tumor progression in prostate cancer (5), non-clear cell renal cell carcinoma (6), hepatocellular carcinoma (HCC) (7), and non-small cell lung cancer (NSCLC) (8). OPN can promote the malignant progression of various cancers by regulating tumor angiogenesis (9), distant metastasis (10–12), maintenance of a stem-like phenotype (13), tumor stromal fibrosis (14), activation of cell proliferation pathways (15, 16), medical treatment resistance (17), and interference with immune function (18–20).

The tumor microenvironment (TME) contains multiple cells that play a crucial role in cancer pathogenesis. There are immune cells, fibroblasts, extracellular matrix, and related cytokines harbored in the TME. In contrast, tumor-associated macrophages (TAMs) are the main component of TME and are considered promising targets for the diagnosis and treatment of cancer. TAMs interact with cancer-associated fibroblasts (CAFs) and other immune components to facilitate the development and progression of cancers.

It was reported that OPN is expressed in activated TAMs and OPN plays an essential role in TAM function during tumorigenesis and tumor progression. But the underlying mechanisms of the OPN in the regulation of TAMs have not been thoroughly investigated. This review summarizes the recent studies of OPN and TAMs and discusses the potential mechanisms for the function of OPN on TAMs.

## OPN and Its Receptors

OPN protein, which is produced by tumor cells, endothelial cells, smooth muscle cells, fibroblasts, and immune cells, is extensively modified after translation. The molecular weight of OPN varies from 44 to 75 kd, depending on the living organ species and cell types (21). The structure, regulation, physiological, and pathological effects of OPN have been well summarized in the recently published reviews, especially for age-related nonalcoholic fatty liver disease, chronic liver disease, cardiac fibrosis, pulmonary fibrosis, and multiple sclerosis (7, 22–25).

**Figure 1:**
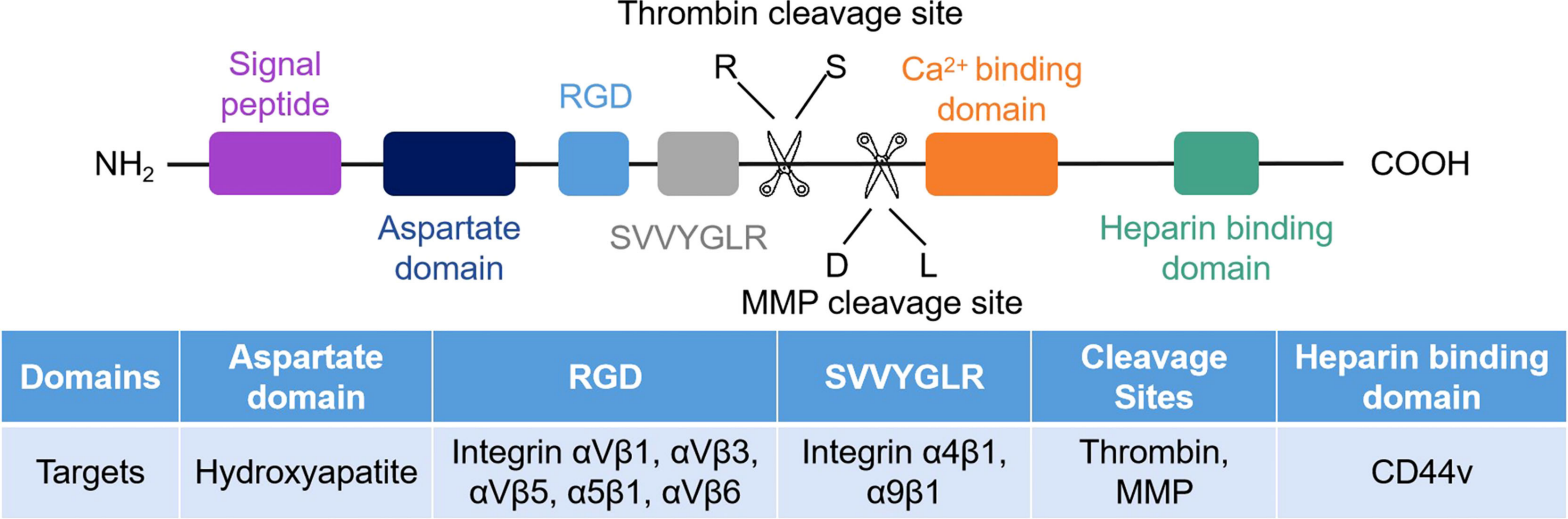
Schematic of the human OPN protein structure. There are seven exons encoded in human OPN protein. CD44 and integrins are the receptors of OPN, and their corresponding binding regions are different.

The structure of OPN consists of the RGD sequence, SVVYGLR sequence, thrombin cleavage site, matrix metalloproteinase (MMP) site, calcium and heparin binding domains (**Figure 1**) (26, 27). Human OPN can form five different isoforms: OPN-a (full length), OPN-b (lacks exon 5), OPN-c (lacks exon 4), OPN-4 (also termed OPN-d, lacks exon 4 and 5), and OPN-5 (the longest isoform, with an extra exon located between canonical exons 3 and 4) (7, 28, 29). The elevated level of OPN-a suggests a poor clinical prognosis in gastric cancer (30). OPN-b resists tumor cell apoptosis in glioma (31). OPN-c is not present in normal breast tissue but is highly expressed in breast cancer and promotes tumor progression independent of traditional prognostic molecules, such as ER, PR, and HER2, as a marker of breast cancer progression (32, 33). OPN-4 and OPN-5 are expressed in esophageal adenocarcinomas and distinct cancer cell lines (34). OPN-5 is expressed higher than OPN-b and OPN-c in normal skin (29). However, the mechanism of OPN-4 or OPN-5 in regulating tumor progression is not fully investigated.

**Figure 2:**
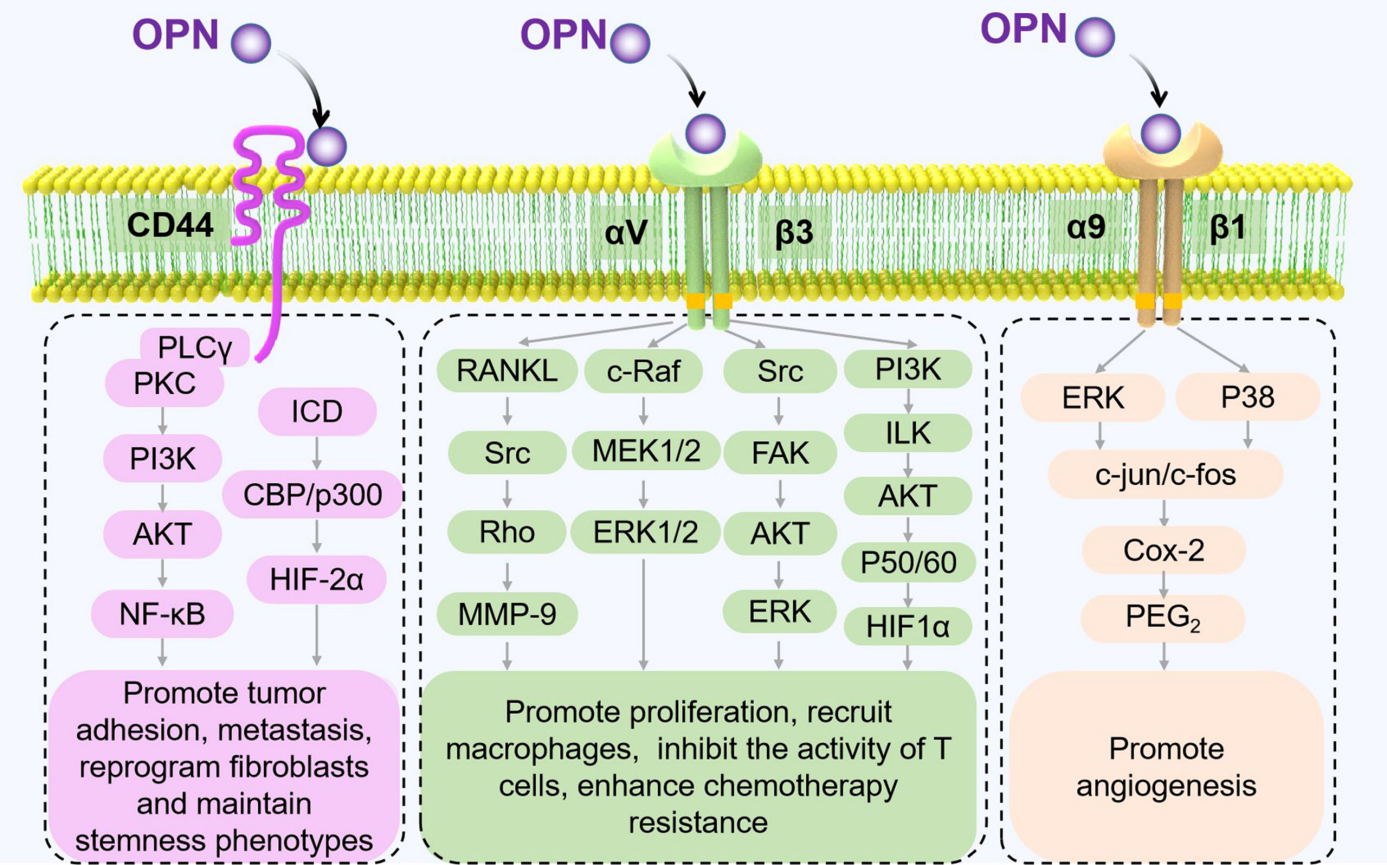
Receptors of OPN and their signaling pathways in tumor progression. The different signaling pathways between OPN and its major receptors, CD44 and integrins, are shown to mediate pathological processes in TME (17, 35–40).

The functions of OPN also vary with different receptors. CD44 and partial integrin proteins (integrins αVβ1, αVβ3, αVβ5, αVβ6, α4β1, α5β1, and α9β1) are known OPN receptors (23). By binding to these receptors, OPN triggers various signaling pathways and regulates tumor progression (**Figure 2**).

CD44 proteins, which form a multifunctional family of single-chain transmembrane glycoproteins, play an essential role in tumor progression and metastasis (41). CD44 isoforms, CD44v6 and v10, are engaged in the interaction of OPN. OPN which was secreted by tumor-associated cells, increases the expression of CD44v6 in colorectal cancer stem cells (CR-CSCs) by activating the PI3K/AKT pathway, thereby promoting the migration and metastasis of CR-CSCs (42). In malignant pleural mesothelioma, OPN transfection significantly increases the adhesion of tumor cells to hyaluronic acid (HA), which acts as a barrier to drugs, resulting in drug resistance of tumor cells to NVB, VP-16, and gemcitabine (GEM) (43).

Integrins play a central role in the interaction with receptors that are involved in cell adhesion and signal transduction. Numerous studies have demonstrated that integrins have multiple functions in tumorigenesis (44). Combined with integrins, particularly αVβ3 and α9β1, OPN could mediate cell–cell and cell–ECM interactions and promote tumor progression (45, 46).

*Integrin αVβ1.* Integrin αVβ1 is highly expressed in mesenchymal cells (MSCs). Further studies have revealed that the expression of C/EBPα and C/EBPβ, which play an important role in promoting adipogenic differentiation, is upregulated in the absence of OPN or the blockade of integrin αVβ1. Therefore, OPN maintains a balance between normal adipogenesis and osteogenesis of MSCs by inhibiting C/EBP activation through integrin αVβ1 (47, 48).

*Integrin αVβ3.* In non-small-cell lung cancer, OPN promotes inhibitor resistance of acquired epidermal growth factor receptor tyrosine kinase (EGFR-TKI) by upregulating integrin αVβ3 expression and activating downstream FAK/AKT and ERK signaling pathways that promote tumor cell proliferation (17).

*Integrin αVβ6.* Integrin αVβ6 binds with OPN on the surface of the porcine Tr cell line (pTr2) to promote the adhesion of pTr2 cells and cationic dependence. Similar to pTr2 cells, porcine uterine epithelial cells (pUE) also bind with OPN through integrin αVβ3 expressed on their surfaces. OPN regulates trophoblast ectodermal cell migration and epithelial cell adhesion by binding with integrin αVβ6 or αVβ3 respectively on their surfaces (49).

*Integrin α4β1.* In rheumatoid arthritis and alcoholic hepatitis models, monocytes and neutrophils recruited by cleaved OPN highly express integrin α4β1 on inflammatory sites by binding with the exposed SLAYGLR motif. In line with this finding, OPN-mediated migration of monocytes and neutrophils is almost entirely inhibited by antibodies against the SLAYGLR motif (M5 antibody) (50, 51). In multiple sclerosis models, OPN increases phosphorylation of IKKβ and activation of the NF-κB pathway in target cells by binding to integrin α4β1 expressed on the surface of target cells (52).

*Integrin α5β1.* The presence of the divalent cation Mn2^+^ and/or phorboester TPA significantly increases the activation of α5β1, which is required for the binding of integrin α5β1 to OPN by RGD motifs (53).

*Integrin α9β1.* The results from melanoma B16F10 mouse model studies revealed that OPN significantly increased the infiltration of CD31^+^ cells and cyclooxygenase subtype 2 (COX-2) positive macrophages in tumor cells. OPN was found to activate the ERK and P38 signaling pathways by binding with integrin α9β1, leading to the expression of COX-2, a key rate-limiting enzyme that regulates prostaglandin synthesis in macrophages (9).

OPN can also interact with some G protein coupled receptors (GPCRs) (e.g., through β2-adrenergic receptors (ARs) to regulate cardiomyocyte fibrosis and bone metabolism). In terms of cardiomyocyte fibrosis, OPN inhibits the expression of cAMP and exchange protein directly activated by cyclic-adenosine monophosphate1 (Epac1), where cAMP is the downstream signaling and major second messenger generated by β2-ARs, and Epac1 is one of the effectors of cAMP that can impede collagen synthesis (54, 55). In bone metabolism-associated processes, isoproterenol (ISO) stimulates sympathetic nervous system tension and causes bone mass loss in WT mice but does not affect OPN-KO mice. However, neutralized extracellular OPN yields limited improvement in ISO-induced bone loss. The mechanism may have two aspects: 1) OPN is the necessary element of ISO-inducing bone metabolism; and 2) intracellular OPN (iOPN) inhibits GPCRs, inhibiting the production of cAMP generated by β2-ARs and cAMP-response element transcription in osteoblasts (56).

## TAMs in Tumor

Solid tumors are *in vivo* three-dimensional organ-like structures consisting of tumor cells and non-malignant stromal cells. Tumor-associated macrophages (TAMs) are the major components of tumor-infiltrating immune cells (57, 58). TAMs are the only colonies of macrophages present in TME. Macrophages in TME could harbor either an activated M1 or an alternatively activated M2 polarization profile by factors in TME (59). Strictly speaking, TAM is not a macrophage classification, which dictates the opposing effects on plasticity or heterogeneity in TME. Current studies have shown that TAMs consist of tissue-resident macrophages and peripheral blood-derived monocytes (60). TAMs can constantly be subjected to transition between M1 and M2, and different phenotypes of macrophages can co-exist in TME (61, 62). Activated M1-like TAMs are characterized as producing reactive oxygen species/reactive nitrogen species and pro-inflammatory cytokines (e.g., interleukin-1β, interleukin-6, and tumor necrosis factor-α) and contributing to the innate immune defense and the role of killing tumor cells. Therefore, activated M1-like TAMs are considered anti-tumor M1 (63). However, M2-like TAMs consist of low efficiency of antigen presentation and promote cancer progression through the production of the immunosuppressive cytokines, such as IL-10 and transforming growth factor-β (TGF-β), which have been identified as the main factor of immunosuppression and the marker of poor prognosis in the tumor microenvironment (64). M2-like TAMs could be defined into four subtypes: M2a, M2b, M2c, and M2d (65). Furthermore, the M2d subset plays a role in immune suppression and pro-tumor, which could be activated by growth factors and cytokines in the TME. Most TAM phenotypes were M2-type macrophages, which facilitate tumor growth and metastasis, tissue remodeling, promotion of angiogenesis, and adaptive immune suppression (66).

Triple-negative breast cancer (TNBC) is famous for its high tumor heterogeneity, which may lead to reduced patient response to medical treatment (67). Tumor heterogeneity is not only related to the cancer cells themselves but also to the immune cells infiltrating the TME. RNA sequencing results revealed that M2-type TAMs are the main constituents of TME in TNBC (68). In these TAMs, the expression of TGF-β1, MS4A6A, CD163, IL8, and PLAUR genes were significantly increased, which are closely related to angiogenesis and epithelial–mesenchymal transition (EMT) (67). Meanwhile, other immunosuppressive cells in the TME, such as T-reg cells, indirectly promote the activation of M2-type macrophages to protect tumor cells from cytotoxic killing and inhibit the immune response (69). Furthermore, TAMs could counteract the anti-tumor effect of tumor infiltrating NK and T cells and exert a synergistic promotion effect on immunosuppressive TME with myeloid-derived suppressor cells, tumor-associated DCs, and neutrophils (70).

## Interactions Between OPN and TAMs

OPN is identified as an immunomodulatory molecule of activated T lymphocytes and is known as early T lymphocyte activation-1 (Eta-1) (71). It functions as a proinflammatory cytokine and chemokine that plays a crucial role in immune cell functions, including T, B, NK, and NKT cells; macrophages; DCs; monocytes; neutrophils; and eosinophils (72–74). OPN, released by tumor cells and TAMs in TME, has been identified as a multifunctional factor in cancer promotion and metastasis in several cancers (21), including breast, stomach, lung, prostate, liver, and colon cancer. It has been revealed that TAMs secrete excess colony-stimulating factor-1 (CSF1) with the help of OPN in hepatocellular carcinoma TME. This process facilitated the recruitment of macrophages and the transformation of the TAMs, which increased the expression of PD-L1 and the immune suppressive microenvironment (18).

The OPN in TAMs is known as TOPN. OPN expressed by myeloid and tumor cells endows tumor immune tolerance by inhibiting CD8 T-cell activation and recruiting inhibitory macrophages (18, 75, 76). Interferon regulatory factor 8 (IRF8) is an important transcription factor in myeloid cells and plays a key role in the development of monocytes and plasmacytoid dendritic cells (77, 78). IRF8 is mostly silent in MDSCs and could directly bind to the SPP1 promoter to inhibit OPN expression (79). In this way, the silencing of IRF8 in MDSCs and tumor cells led to increased expression of OPN. When OPN was overexpressed, it inhibited IFNγ production in mouse CD8 T cells, thereby reducing the antitumor activity of CD8 T cells (75). The results reported by Li et al. indicated that OPN secreted by TAMs upregulated PD-L1 expression through the NF-κB/P65 pathway in an NSCLC mouse model, and TOPN was positively correlated with PD-L1 expression in NSCLC patients (19). FAP^+^ fibroblasts and SPP1^+^ macrophages are the major components of TME in colorectal cancer, which are characterized as major contributors to the desmoplastic tumor structure and immunotherapy resistance against PD-L1 in colorectal cancer (80). Some pieces of evidence have demonstrated that OPN infiltrating macrophages facilitated tumor cell survival and angiogenesis in glioblastoma multiforme (GBM) (81). In TNBC, TOPN released by TAMs can also regulate tumor metastasis. TAMs release cytokines including OPN, CCL7, CCL19, CXCL7, NRG3, HGF, and TGF-β3 and regulate tumor metastasis (82). Understanding the link between TOPN and TAMs in TME and their various functions in tumor progression, angiogenesis, and stromal remodeling may provide a novel target for cancer treatment. Moreover, OPN secreted from tumor cells is one of the crucial drivers of TAMs recruitment and polarization, tumor angiogenesis, and tumor fibrosis promotion, which will be discussed later.

**Figure 3:**
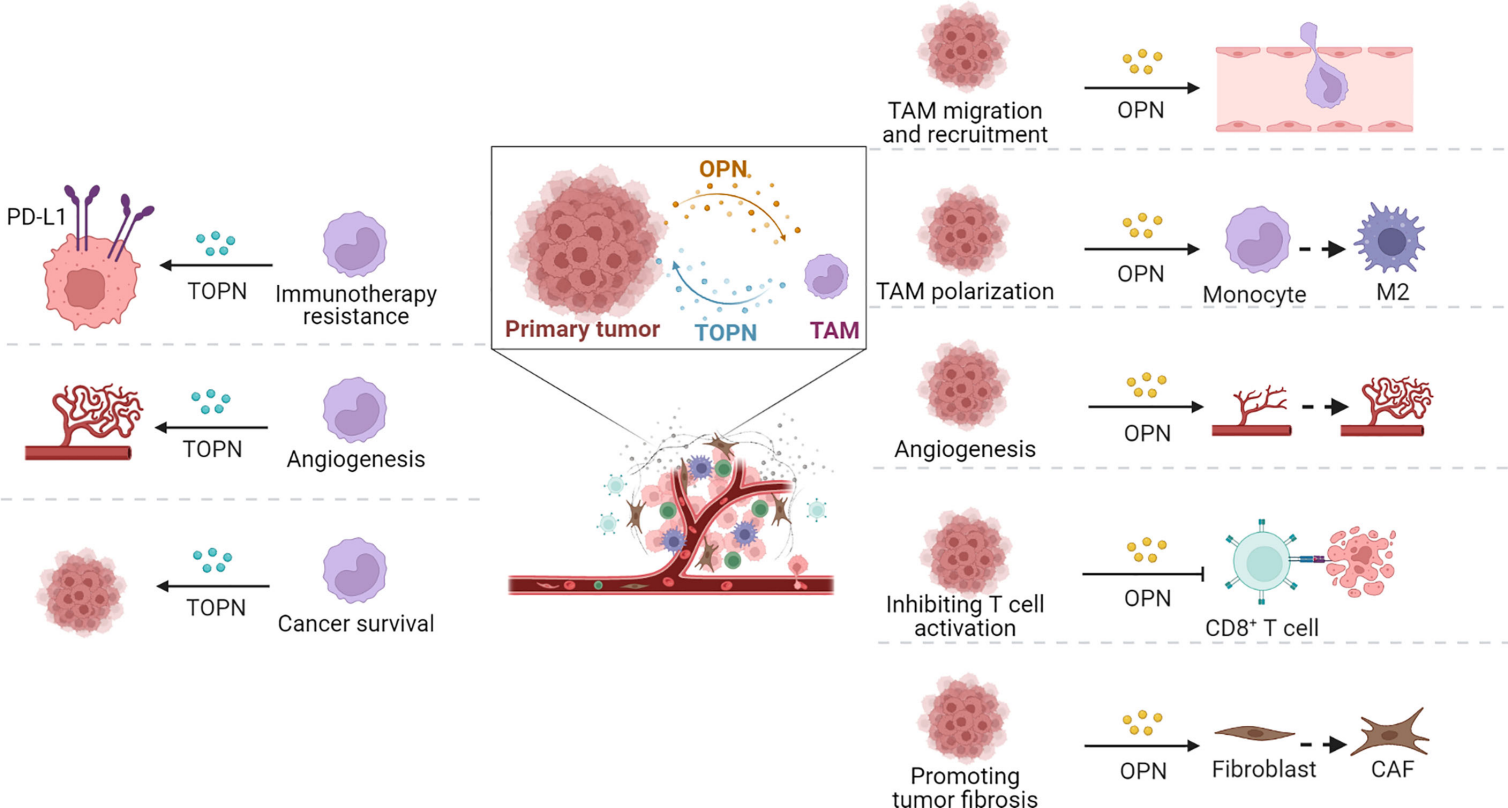
Functions of OPN and TOPN secreted by tumor cells and macrophages separately in TME. OPN played roles in tumor progression through TAMs (right part) and the effect of macrophage-derived OPN, termed TOPN, functioned in tumor tissue summarized (left part).

Given the supporting functions of TME for tumor cells, an appropriate TME is important to the development and progression of tumors, except for the malignant characteristics of tumor cells themselves. The TME plays an important role in the recruitment of immunosuppressive cells, education or destruction of normal stromal cells and vascular endothelial cells, and metastasis to distant areas to escape immune surveillance of the host. Numerous studies have confirmed that tumor-associated OPN participates in TAMs migration and recruitment, polarization, tumor fibrosis, tumor angiogenesis, and immune homeostasis (**Figure 3**).

## OPN Promotes Tumor Progression by Acting on TAMs

Various cancer models have confirmed that OPN can regulate tumor progression by recruiting macrophages. In tumor tissues, OPN, as a major chemokine, can regulate macrophage migration by interacting with integrin αVβ5, CD44, GPCR, or the CSF1–CSF1R axis. With the accumulation of macrophages in TME, they are educated to become M2-type TAMs, and further promote tumor stromal fibrosis by secreting TGF-β or platelet-derived growth factors (PDGFs). Recently, single-cell RNA sequencing (scRNA-seq) verified that the cluster of SPP1^+^ TAMs can also be active CAFs. Meanwhile, in the presence of OPN, TAMs promote angiogenesis through JAK/STAT3, NF-κB, and ERK/p38 signaling pathways.

### OPN Facilitates the Migration and Recruitment of TAMs

OPN can act as a chemotaxin for macrophages and is involved in the control of macrophages migration and recruitment. It has been well demonstrated both *in vivo* and *in vitro* that integrins, CD44, chemokines, GPCRs are intimately involved in the regulation of macrophage migration, which process can be regulated by OPN.

In hepatocellular carcinoma (HCC), tumor-released OPN can stimulate macrophages to secrete CSF1 through the PI3K–ATK–p65 signaling pathway and then induce infiltration of macrophages. Excessive macrophages play an important role in the recurrence of HCC (18, 83). In GBM patients, high OPN expression is positively correlated with TAMs infiltration and tumor progression and negatively correlated with survival prognosis. In line with the studies in humans, depletion of OPN in mice resulted in reduced TAM infiltration and increased the survival rate of mice with GL261 GBM (76). The mechanism of these phenotypes is associated with integrin αVβ5, which is highly expressed in GBM-infiltrated TAMs (76). Zhu et al. (84) found that OPN and GPCRs are related to the migration of macrophages. Their study indicated that in the presence of N-formyl-methionyl-leucyl-phenylalanine (fMLP), which can activate GPCRs, WT macrophages migrate nearly twice as much as OPN^−/−^ macrophages. CD44^−/−^ macrophages exhibit the lowest migration rate. With the supplement of exogenous OPN, the migration of OPN^−/−^ macrophages can be restored in an OPN dose-dependent manner. However, exogenous OPN did not rescue the impaired chemotaxis of CD44^−/−^ macrophages to fMLP. These results indicate that CD44 is the necessary factor in the GPCRs-mediated migration and that iOPN could modulate the CD44 activity.

However, in the spontaneous tumor model of breast cancer, although OPN is highly expressed in the tumor tissues of transgenic mice, the incidence of spontaneous tumor and tumor volume are independent of the presence of OPN. Unexpectedly, the number of macrophages in tumors of WT and OPN-KO mice has been found to be independent of the OPN genotype (85). The authors speculated that the lack of OPN may lead to compensatory mechanisms that promote tumor progression or the dependence of spontaneous tumor models on OPN, which may be different from orthotopic implanted tumor models. It has been demonstrated in another study that OPN-KO mice showed a reduction of infiltrating macrophages in tumor tissue, while OPN-KO treatment has little effect on infiltrating macrophages in normal tissue (9). The above studies indicated that OPN can be used as a specific factor to regulate the roles of macrophages in the infiltration of tumor tissues.

### OPN Induces and Maintains the Alternative M2 Activation of TAMs

Alternatively, the activated M2 polarization profile of TAMs is considered an indispensable component of TME. Although most literature has confirmed that OPN has a recruitment effect on macrophages, this is inconsistent with the effects of OPN on the TAM polarization (18, 76). OPN originating from tumor cells can induce the monocytes to undergo alternative M2 activation. The percentage of M2 macrophages was significantly increased when the human monocyte cell line U937 was treated with OPN-positive conditioned medium of the human gastric cancer cell line AGS. While a mixture of co-cultured OPN^+^ AGS and U937 cells was inoculated into the back skin of nude mice, the xenografts from the mixture showed faster growth and correlated with poorer survival compared with the inoculation of OPN^+^ AGS cells alone (86). In HCC patients, the expression of OPN was positively correlated with the infiltration of TAMs in tumor tissues. By analyzing the numbers of tumor-infiltrating immune cells and profiles of chemically induced liver tumors from WT and OPN-KO mice, OPN derived from host and HCC cells was found to stimulate macrophages to secrete CSF-1 and then activate the CSF1-CSF1R axis of macrophages to promote macrophage chemotaxis and M2-like polarization in HCC cells (18). These studies show that OPN participates in the process of M2-like macrophage polarization and maintains an M2-like macrophage phenotype.

However, Wei et al. (76) have indicated that OPN maintained the genetic characteristics and phenotype of M2 TAMs but did not induce TAM polarization (76). In this study, the healthy donors were treated with various concentrations of recombinant OPN protein. After that, the representative markers of M2-like macrophages were examined. Interestingly, the markers of M2-like macrophages did not respond to the treatment with recombinant OPN protein, and the number of M2-like macrophages did not significantly change (76).

Tissue macrophages have long been thought to develop from monocytes that enter tissues after circulating in the blood. However, with the development of molecular technology and the establishment of new animal models, this concept is increasingly questioned (87, 88). Studies on mice and humans have shown that macrophages in tissues can be divided into tissue-resident macrophages (TRMs) and blood-derived macrophages according to their sources and physiological characteristics, and the proportions of blood-derived macrophages and TRMs in different organs are different. Microglia in the brain are derived from primitive macrophages in the embryonic yolk sac, and erythrocytic myeloid progenitor cells (EMPs) in the yolk sac are the primary source of Kupffer cells (89–92). In tumor tissues, macrophages are derived not only from peripheral blood but also from a group of TRMs that are involved in the formation of TAMs. In the above experiments, macrophages were considered a single population without considering different sources of macrophages. Not only is the polarization effect of OPN on the blood source and TRM unclear, but also the polarization effect of OPN on TRM has different origins. Although lacking experimental evidence, the reported different effects of OPN on TAM polarization between different mouse models may be possible due to the difference in TRMs.

It has been reported that PD-L1 is downregulated in OPN deficient macrophages and the markers of M1-like macrophages exist predominately rather than M2-like macrophages (93). Meanwhile, OPN in HCC promoted PD-L1 expression in macrophages by activating the CSF1-CSF1R pathway. The combination of anti-PD-L1 antibody and CSF1R inhibitor could promote the infiltration of CD8^+^T cells and reduce the location of TAMs, which are beneficial to the HCC therapeutic effect of anti-PD-L1 antibody (18).

### OPN Promote Tumor Fibrosis *via* TAMs

OPN can promote tumor fibrosis through its chemotactic effect on macrophages and activation of cancer-associated fibroblasts (CAFs) in TME (14, 94–96). TAMs and CAFs can cross-talk in the TME: CAFs can secrete chemokines to attract monocytes into the tumor microenvironment and differentiate into TAMs; TAMs can promote fibroblast activation by secreting TGF-β or promote fibroblast proliferation by secreting PDGFs (97). Single-cell RNA sequencing (scRNA-seq) analysis from colorectal cancer patients showed that SPP1^+^ TAMs expressing syndecan-2 (SDC2) were more likely to interact with CAFs expressing MMP-2 through the combination of SDC2 and MMP-2 to promote the activation of CAFs and tumor tissue fibrosis. Notably, SPP1^+^ TAMs were resistant to CSF1R blockade in a mouse model, and high infiltration of SPP1^+^ TAMs in colon cancer patients had a poor prognosis (96).

Although TAMs have a significant feature of promoting fibrosis (such as collagen synthesis and deposition, etc.), studies on hepatic fibrosis disease models have found that macrophages play different or opposite roles in different stages of fibrosis progression (98, 99). In the case of inflammatory injury, clearance of macrophages helps alleviate the accumulation of abnormal collagen in the injured liver. Unexpectedly, during the recovery phase, macrophages promoted matrix degradation and absorption. After macrophage depletion, the proportion of Sirius red-staining positive collagen matrix in the liver increases from 1% to more than 3% (98). In a CCL4-induced hepatic fibrosis mouse model, the M1-type macrophages not only had a therapeutic effect on liver fibrosis by increasing the apoptosis of hepatic stellate cells but also recruited more endogenous anti-fibrosis macrophages into the liver by producing chemokines CCL2 and CCL3 (99). Since TAMs are highly heterogenic (100), OPN may play different roles in tumor fibrosis depending on the composition of TAMs in the tumor.

### OPN Promotes Tumor Angiogenesis Through TAMs

Angiogenesis plays a vital role in promoting malignant tumor growth, diffusion, and metastasis. Numerous studies have indicated that TAMs are the crucial factors in regulating tumor angiogenesis (60, 101). TAMs can secrete pro-angiogenic growth factors and release MMPs to promote the degradation of extracellular matrix around blood vessels and facilitate the extension of tumor blood vessels (102).

A few studies have reported that OPN can promote tumor-associated angiogenesis by regulating macrophages with different phenotypes. Immunohistochemistry results of tumor tissue indicated that GBM-related macrophages express metalloprotease-disintegrin 8 (ADAM8), which is associated with invasive and poor prognosis (103–105). It has been reported that the supernatant of ADAM8 overexpressed macrophages can induce human umbilical vein endothelial cells (HUVEC) to form more tube-like structures than the ADAM8 deficient group. However, ADAM8 has no correlation with the polarization of macrophages (106). Nevertheless, the expression of OPN is reduced in ADAM8-deficient macrophages (105). Finally, they found that OPN regulates the angiogenesis of ADAM8-deficient macrophages through JAK/STAT3 and NF-κB signaling pathways (105). In melanoma, the expression of COX-2 in macrophages and the angiogenesis capacity of HUVEC cells were enhanced through an ERK/p38-dependent pathway which was regulated by the OPN secreted by tumor cells (9).

OPN can also promote tumor angiogenesis in a TAM-independent manner. OPN could promote endothelial cell proliferation and activate tumor cells to secrete VEGF. OPN residues in tumors bind to CD44 and integrin receptors to mediate NF-κB, PI3K/Akt, VEGF, uPA, and MMPs to promote endothelial cell proliferation (107, 108). Moreover, OPN regulates the proliferation and growth of muscle-derived angiogenic progenitor cells (MDPCs) through the PI3K/Akt pathway (109). In the breast cancer mouse model, exogenous and tumor-derived OPN can promote VEGF expression and tumor angiogenesis by activating the Brk/NF-κB/ATF-4 signaling pathway (110). However, in the neuroblastoma mouse model, OPN promotes intratumor angiogenesis by stimulating vascular endothelial cell migration (111).

## Conclusions and Perspective

OPN, as a secreted protein, has complicated biological functions and plays an important role in the regulation of tumorigenesis, anti-tumor immunity, and modulation of TME. The effects of OPN on immune regulation have been confirmed in diverse diseases, such as inflammatory and autoimmune disease models. It is not surprising that the neutralizing antibodies of OPN have been proven to alleviate various inflammatory-mediated diseases, such as osteoporosis, hepatitis, and arthritis. Furthermore, some monoclonal antibodies to OPN have been used in therapy strategies in the context of cancer. For example, anti-OPN antibodies retard the growth and reduce metastasis of breast cancer in mice (112). Unfortunately, the detailed mechanisms of OPN function in TME have not been fully developed. Simply neutralizing or completely depleting their activities is unlikely to be an optimal or effective approach (113–115).

Recently, the potential of programmed macrophage subsets has been explored, while OPN participating in the redefinition of TAMs subpopulations and functions in the steady state would be a promising tumor immune treatment strategy. However, we believe that the following issues should be considered when developing OPN or TAM-targeted strategies: Firstly, OPN is a multifunctional factor that plays a cell-specific role in inflammation, immunity, and tissue repair, and it has various variants with different activities (116). In the TME, macrophages are the major constituent cell population, but not the only one. The depletion of TAMs may impair the antitumor effect of TME or compensatory stimulation of the proliferation of other cells with immunosuppressive function (i.e., MDSC) and aggravate tumor progression. Secondly, some reports suggest that OPN exhibits anti-tumor characteristics under certain circumstances: 1) OPN deficiency in squamous cell carcinoma mouse models leads to accelerated tumor growth (117). In intrahepatic cholangiocarcinoma, the high expression level of OPN in tumors indicates better overall survival and decreased lymph node metastasis (118), 2) OPN-deficient macrophages exhibit impaired antitumor cytotoxicity (117), and 3) stromal-derived OPN enhances NK cell infiltration into the prostate tumor in the genetically modified mice (119). Thirdly, both OPN and TAMs are phenotypically and functionally heterogeneous (120, 121), and there are still many gaps in our understanding of the effects of OPN on TAMs. Therefore, further in-depth studies are warranted to understand the underlying mechanisms of OPN and TAMs in tumorigenesis and tumor progression, which may offer new hope for future cancer treatments.

## Author Contributions

YT, Y-GY, and WL conceptualized the study. YT and LZ performed the literature search, data analysis and drafted the paper. Y-GY and WL critically revised the work. All authors listed have made a substantial, direct, and intellectual contribution to the work and approved it for publication.

## Funding

This work was supported by grants from the National key R & D program of China (2021YFA1100700) and the Natural Science Foundation of Jilin Province, China (20200201191JC).

## Conflict of Interest

The authors declare that the research was conducted in the absence of any commercial or financial relationships that could be construed as a potential conflict of interest.

## Publisher’s Note

All claims expressed in this article are solely those of the authors and do not necessarily represent those of their affiliated organizations, or those of the publisher, the editors and the reviewers. Any product that may be evaluated in this article, or claim that may be made by its manufacturer, is not guaranteed or endorsed by the publisher.

## References

[1] Sodek J Ganss B McKee MD. Osteopontin. Crit Rev Oral Biol Med (2000) 11(3):279–303. doi: 10.1177/10454411000110030101 11021631

[2] Ashkar S Weber GF Panoutsakopoulou V Sanchirico ME Jansson M Zawaideh S. Eta-1 (Osteopontin): An Early Component of Type-1 (Cell-Mediated) Immunity. Science (2000) 287(5454):860–4. doi: 10.1126/science.287.5454.860 10657301

[3] Rao G Wang H Li B Huang L Xue D Wang X. Reciprocal Interactions Between Tumor-Associated Macrophages and CD44-Positive Cancer Cells *via* Osteopontin/CD44 Promote Tumorigenicity in Colorectal Cancer. Clin Cancer Res (2013) 19(4):785–97. doi: 10.1158/1078-0432.CCR-12-2788 23251004

[4] Qin X Yan M Wang X Xu Q Wang X Zhu X. Cancer-Associated Fibroblast-Derived IL-6 Promotes Head and Neck Cancer Progression *via* the Osteopontin-NF-Kappa B Signaling Pathway. Theranostics (2018) 8(4):921–40. doi: 10.7150/thno.22182 PMC5817102 29463991

[5] Wisniewski T Zyromska A Makarewicz R Zekanowska E. Osteopontin And Angiogenic Factors As New Biomarkers Of Prostate Cancer. Urol J (2019) 16(2):134–40. doi: 10.22037/uj.v0i0.4282 30178447

[6] Armstrong AJ Nixon AB Carmack A Yang Q Eisen T Stadler WM. Angiokines Associated With Targeted Therapy Outcomes in Patients With Non-Clear Cell Renal Cell Carcinoma. Clin Cancer Res (2021) 27(12):3317–28. doi: 10.1158/1078-0432.CCR-20-4504 33593885

[7] Song Z Chen W Athavale D Ge X Desert R Das S. Osteopontin Takes Center Stage in Chronic Liver Disease. Hepatology (2021) 73(4):1594–608. doi: 10.1002/hep.31582 PMC8106357 32986864

[8] Shi L Hou J Wang L Fu H Zhang Y Song Y. Regulatory Roles of Osteopontin in Human Lung Cancer Cell Epithelial-to-Mesenchymal Transitions and Responses. Clin Transl Med (2021) 11(7):e486. doi: 10.1002/ctm2.486 34323425 PMC8265167

[9] Kale S Raja R Thorat D Soundararajan G Patil TV Kundu GC. Osteopontin Signaling Upregulates Cyclooxygenase-2 Expression in Tumor-Associated Macrophages Leading to Enhanced Angiogenesis and Melanoma Growth *via* Alpha9beta1 Integrin. Oncogene (2014) 33(18):2295–306. doi: 10.1038/onc.2013.184 23728342

[10] Moorman HR Poschel D Klement JD Lu C Redd PS Liu K. Osteopontin: A Key Regulator of Tumor Progression and Immunomodulation. Cancers (Basel) (2020) 12(11):3379. doi: 10.3390/cancers12113379 PMC7698217 33203146

[11] Liu K Hu H Jiang H Zhang H Gong S Wei D. RUNX1 Promotes MAPK Signaling to Increase Tumor Progression and Metastasis *via* OPN in Head and Neck Cancer. Carcinogenesis (2021) 42(3):414–22. doi: 10.1093/carcin/bgaa116 33175152

[12] Raineri D Dianzani C Cappellano G Maione F Baldanzi G Iacobucci I. Osteopontin Binds ICOSL Promoting Tumor Metastasis. Commun Biol (2020) 3(1):615. doi: 10.1038/s42003-020-01333-1 33106594 PMC7588454

[13] Nallasamy P Nimmakayala RK Karmakar S Leon F Seshacharyulu P Lakshmanan I. Pancreatic Tumor Microenvironment Factor Promotes Cancer Stemness *via* SPP1-CD44 Axis. Gastroenterology (2021) 161(6):1998–2013.e7. doi: 10.1053/j.gastro.2021.08.023 34418441 PMC10069715

[14] Weber CE Kothari AN Wai PY Li NY Driver J Zapf MA. Osteopontin Mediates an MZF1-TGF-Beta1-Dependent Transformation of Mesenchymal Stem Cells Into Cancer-Associated Fibroblasts in Breast Cancer. Oncogene (2015) 34(37):4821–33. doi: 10.1038/onc.2014.410 PMC4476970 25531323

[15] Huang L Song F Sun H Zhang L Huang C. IRX5 Promotes NF-kappaB Signalling to Increase Proliferation, Migration and Invasion *via* OPN in Tongue Squamous Cell Carcinoma. J Cell Mol Med (2018) 22(8):3899–910. doi: 10.1111/jcmm.13664 PMC6050492 29761910

[16] Peraramelli S Zhou Q Zhou Q Wanko B Zhao L Nishimura T. Thrombin Cleavage of Osteopontin Initiates Osteopontin's Tumor-Promoting Activity. J Thromb Haemost (2022) 20(5):1256–70. doi: 10.1111/jth.15663 PMC9289821 35108449

[17] Fu Y Zhang Y Lei Z Liu T Cai T Wang A. Abnormally Activated OPN/integrin Alphavbeta3/FAK Signalling is Responsible for EGFR-TKI Resistance in EGFR Mutant non-Small-Cell Lung Cancer. J Hematol Oncol (2020) 13(1):169. doi: 10.1186/s13045-020-01009-7 33287873 PMC7720454

[18] Zhu Y Yang J Xu D Gao XM Zhang Z Hsu JL. Disruption of Tumour-Associated Macrophage Trafficking by the Osteopontin-Induced Colony-Stimulating Factor-1 Signalling Sensitises Hepatocellular Carcinoma to Anti-PD-L1 Blockade. Gut (2019) 68(9):1653–66. doi: 10.1136/gutjnl-2019-318419 30902885

[19] Li Y Liu H Zhao Y Yue D Chen C Li C. Tumor-Associated Macrophages (TAMs)-Derived Osteopontin (OPN) Upregulates PD-L1 Expression and Predicts Poor Prognosis in non-Small Cell Lung Cancer (NSCLC). Thorac Cancer (2021) 12(20):2698–709. doi: 10.1111/1759-7714.14108 PMC8520804 34423566

[20] Lu C Liu Z Klement JD Yang D Merting AD Poschel D. WDR5-H3K4me3 Epigenetic Axis Regulates OPN Expression to Compensate PD-L1 Function to Promote Pancreatic Cancer Immune Escape. J Immunother Cancer (2021) 9(7):e002624. doi: 10.1136/jitc-2021-002624 34326167 PMC8323468

[21] Icer MA Gezmen-Karadag M. The Multiple Functions and Mechanisms of Osteopontin. Clin Biochem (2018) 59:17–24. doi: 10.1016/j.clinbiochem.2018.07.003 30003880

[22] Gomez-Santos B Saenz de Urturi D Nunez-Garcia M Gonzalez-Romero F Buque X Aurrekoetxea I. Liver Osteopontin is Required to Prevent the Progression of Age-Related Nonalcoholic Fatty Liver Disease. Aging Cell (2020) 19(8):e13183. doi: 10.1111/acel.13183 32638492 PMC7431823

[23] Abdelaziz Mohamed I Gadeau AP Hasan A Abdulrahman N Mraiche F. Osteopontin: A Promising Therapeutic Target in Cardiac Fibrosis. Cells (2019) 8(12):1558. doi: 10.3390/cells8121558 PMC6952988 31816901

[24] Hou J Ji J Chen X Cao H Tan Y Cui Y. Alveolar Epithelial Cell-Derived Sonic Hedgehog Promotes Pulmonary Fibrosis Through OPN-Dependent Alternative Macrophage Activation. FEBS J (2021) 288(11):3530–46. doi: 10.1111/febs.15669 33314622

[25] Clemente N Comi C Raineri D Cappellano G Vecchio D Orilieri E. Role of Anti-Osteopontin Antibodies in Multiple Sclerosis and Experimental Autoimmune Encephalomyelitis. Front Immunol (2017) 8:321. doi: 10.3389/fimmu.2017.00321 28386258 PMC5362623

[26] Venables JP. Aberrant and Alternative Splicing in Cancer. Cancer Res (2004) 64(21):7647–54. doi: 10.1158/0008-5472.CAN-04-1910 15520162

[27] Maeda N Maenaka K. The Roles of Matricellular Proteins in Oncogenic Virus-Induced Cancers and Their Potential Utilities as Therapeutic Targets. Int J Mol Sci (2017) 18(10):2198. doi: 10.3390/ijms18102198 PMC5666879 29065446

[28] Anborgh PH Mutrie JC Tuck AB Chambers AF. Pre- and Post-Translational Regulation of Osteopontin in Cancer. J Cell Commun Signal (2011) 5(2):111–22. doi: 10.1007/s12079-011-0130-6 PMC3088790 21516514

[29] Chou CF Huang CC Bin Dabil N Chang PL. Assessing SPP1/Osteopontin (OPN) Splice Variants and Their Association to Nonmelanoma Skin Cancer by Absolute Quantification: Identification of OPN-5 Subvariants and Their Protein Coding Potential. Cancer Invest (2021) 39(6-7):559–70. doi: 10.1080/07357907.2021.1933015 34043476

[30] Hao C Cui Y Lane J Jia S Ji J Jiang WG. Distinctive Prognostic Value and Cellular Functions of Osteopontin Splice Variants in Human Gastric Cancer. Cells (2021) 10(7):1820. doi: 10.3390/cells10071820 34359989 PMC8305399

[31] Yan W Qian C Zhao P Zhang J Shi L Qian J. Expression Pattern of Osteopontin Splice Variants and its Functions on Cell Apoptosis and Invasion in Glioma Cells. Neuro Oncol (2010) 12(8):765–75. doi: 10.1093/neuonc/noq006 PMC2940675 20511184

[32] Zduniak K Ziolkowski P Ahlin C Agrawal A Agrawal S Blomqvist C. Nuclear Osteopontin-C is a Prognostic Breast Cancer Marker. Br J Cancer (2015) 112(4):729–38. doi: 10.1038/bjc.2014.664 PMC4333500 25625274

[33] Walaszek K Lower EE Ziolkowski P Weber GF. Breast Cancer Risk in Premalignant Lesions: Osteopontin Splice Variants Indicate Prognosis. Br J Cancer (2018) 119(10):1259–66. doi: 10.1038/s41416-018-0228-1 PMC6251032 30353046

[34] Silva GR Mattos DS Bastos ACF Viana B Brum MCM Ferreira LB. Osteopontin-4 and Osteopontin-5 Splice Variants are Expressed in Several Tumor Cell Lines. Mol Biol Rep (2020) 47(10):8339–45. doi: 10.1007/s11033-020-05867-9 33006711

[35] Pietras A Katz AM Ekstrom EJ Wee B Halliday JJ Pitter KL. Osteopontin-CD44 Signaling in the Glioma Perivascular Niche Enhances Cancer Stem Cell Phenotypes and Promotes Aggressive Tumor Growth. Cell Stem Cell (2014) 14(3):357–69. doi: 10.1016/j.stem.2014.01.005 PMC3999042 24607407

[36] Desai B Rogers MJ Chellaiah MA. Mechanisms of Osteopontin and CD44 as Metastatic Principles in Prostate Cancer Cells. Mol Cancer (2007) 6:18. doi: 10.1186/1476-4598-6-18 17343740 PMC1828067

[37] Zhao H Chen Q Alam A Cui J Suen KC Soo AP. The Role of Osteopontin in the Progression of Solid Organ Tumour. Cell Death Dis (2018) 9(3):356. doi: 10.1038/s41419-018-0391-6 29500465 PMC5834520

[38] Robertson BW Bonsal L Chellaiah MA. Regulation of Erk1/2 Activation by Osteopontin in PC3 Human Prostate Cancer Cells. Mol Cancer (2010) 9:260. doi: 10.1186/1476-4598-9-260 20868520 PMC3098013

[39] Raja R Kale S Thorat D Soundararajan G Lohite K Mane A. Hypoxia-Driven Osteopontin Contributes to Breast Tumor Growth Through Modulation of HIF1alpha-Mediated VEGF-Dependent Angiogenesis. Oncogene (2014) 33(16):2053–64. doi: 10.1038/onc.2013.171 23728336

[40] Kale S Raja R Thorat D Soundararajan G Patil TV Kundu GC. Osteopontin Signaling Upregulates Cyclooxygenase-2 Expression in Tumor-Associated Macrophages Leading to Enhanced Angiogenesis and Melanoma Growth *via* Alpha9beta1 Integrin. Oncogene (2015) 34(42):5408–10. doi: 10.1038/onc.2015.315 26473949

[41] Morath I Hartmann TN Orian-Rousseau V. CD44: More Than a Mere Stem Cell Marker. Int J Biochem Cell Biol (2016) 81(Pt A):166–73. doi: 10.1016/j.biocel.2016.09.009 27640754

[42] Todaro M Gaggianesi M Catalano V Benfante A Iovino F Biffoni M. CD44v6 is a Marker of Constitutive and Reprogrammed Cancer Stem Cells Driving Colon Cancer Metastasis. Cell Stem Cell (2014) 14(3):342–56. doi: 10.1016/j.stem.2014.01.009 24607406

[43] Tajima K Ohashi R Sekido Y Hida T Nara T Hashimoto M. Osteopontin-Mediated Enhanced Hyaluronan Binding Induces Multidrug Resistance in Mesothelioma Cells. Oncogene (2010) 29(13):1941–51. doi: 10.1038/onc.2009.478 20101228

[44] Cooper J Giancotti FG. Integrin Signaling in Cancer: Mechanotransduction, Stemness, Epithelial Plasticity, and Therapeutic Resistance. Cancer Cell (2019) 35(3):347–67. doi: 10.1016/j.ccell.2019.01.007 PMC6684107 30889378

[45] Yokosaki Y Tanaka K Higashikawa F Yamashita K Eboshida A. Distinct Structural Requirements for Binding of the Integrins Alphavbeta6, Alphavbeta3, Alphavbeta5, Alpha5beta1 and Alpha9beta1 to Osteopontin. Matrix Biol (2005) 24(6):418–27. doi: 10.1016/j.matbio.2005.05.005 16005200

[46] Kahles F Findeisen HM Bruemmer D. Osteopontin: A Novel Regulator at the Cross Roads of Inflammation, Obesity and Diabetes. Mol Metab (2014) 3(4):384–93. doi: 10.1016/j.molmet.2014.03.004 PMC4060362 24944898

[47] Rosen ED MacDougald OA. Adipocyte Differentiation From the Inside Out. Nat Rev Mol Cell Biol (2006) 7(12):885–96. doi: 10.1038/nrm2066 17139329

[48] Chen Q Shou P Zhang L Xu C Zheng C Han Y. An Osteopontin-Integrin Interaction Plays a Critical Role in Directing Adipogenesis and Osteogenesis by Mesenchymal Stem Cells. Stem Cells (2014) 32(2):327–37. doi: 10.1002/stem.1567 PMC3961005 24123709

[49] Erikson DW Burghardt RC Bayless KJ Johnson GA. Secreted Phosphoprotein 1 (SPP1, Osteopontin) Binds to Integrin Alpha V Beta 6 on Porcine Trophectoderm Cells and Integrin Alpha V Beta 3 on Uterine Luminal Epithelial Cells, and Promotes Trophectoderm Cell Adhesion and Migration. Biol Reprod (2009) 81(5):814–25. doi: 10.1095/biolreprod.109.078600 19571258

[50] Yamamoto N Sakai F Kon S Morimoto J Kimura C Yamazaki H. Essential Role of the Cryptic Epitope SLAYGLR Within Osteopontin in a Murine Model of Rheumatoid Arthritis. J Clin Invest (2003) 112(2):181–8. doi: 10.1172/JCI17778 PMC164290 12865407

[51] Banerjee A Lee JH Ramaiah SK. Interaction of Osteopontin With Neutrophil Alpha(4)Beta(1) and Alpha(9)Beta(1) Integrins in a Rodent Model of Alcoholic Liver Disease. Toxicol Appl Pharmacol (2008) 233(2):238–46. doi: 10.1016/j.taap.2008.08.008 18778724

[52] Steinman L. A Molecular Trio in Relapse and Remission in Multiple Sclerosis. Nat Rev Immunol (2009) 9(6):440–7. doi: 10.1038/nri2548 19444308

[53] Barry ST Ludbrook SB Murrison E Horgan CM. A Regulated Interaction Between Alpha5beta1 Integrin and Osteopontin. Biochem Biophys Res Commun (2000) 267(3):764–9. doi: 10.1006/bbrc.1999.2032 10673366

[54] Pollard CM Desimine VL Wertz SL Perez A Parker BM Maning J. Deletion of Osteopontin Enhances Beta(2)-Adrenergic Receptor-Dependent Anti-Fibrotic Signaling in Cardiomyocytes. Int J Mol Sci (2019) 20(6):1396. doi: 10.3390/ijms20061396 PMC6470638 30897705

[55] Surinkaew S Aflaki M Takawale A Chen Y Qi XY Gillis MA. Exchange Protein Activated by Cyclic-Adenosine Monophosphate (Epac) Regulates Atrial Fibroblast Function and Controls Cardiac Remodelling. Cardiovasc Res (2019) 115(1):94–106. doi: 10.1093/cvr/cvy173 30016400 PMC6302269

[56] Nagao M Feinstein TN Ezura Y Hayata T Notomi T Saita Y. Sympathetic Control of Bone Mass Regulated by Osteopontin. Proc Natl Acad Sci U.S.A. (2011) 108(43):17767–72. doi: 10.1073/pnas.1109402108 PMC3203767 21990347

[57] Morrison C. Immuno-Oncologists Eye Up Macrophage Targets. Nat Rev Drug Discovery (2016) 15(6):373–4. doi: 10.1038/nrd.2016.111 27245386

[58] Long KB Collier AI Beatty GL. Macrophages: Key Orchestrators of a Tumor Microenvironment Defined by Therapeutic Resistance. Mol Immunol (2019) 110:3–12. doi: 10.1016/j.molimm.2017.12.003 29273393 PMC6008174

[59] Wu K Lin K Li X Yuan X Xu P Ni P. Redefining Tumor-Associated Macrophage Subpopulations and Functions in the Tumor Microenvironment. Front Immunol (2020) 11:1731. doi: 10.3389/fimmu.2020.01731 32849616 PMC7417513

[60] Larionova I Tuguzbaeva G Ponomaryova A Stakheyeva M Cherdyntseva N Pavlov V. Tumor-Associated Macrophages in Human Breast, Colorectal, Lung, Ovarian and Prostate Cancers. Front Oncol (2020) 10:566511. doi: 10.3389/fonc.2020.566511 33194645 PMC7642726

[61] Boutilier AJ Elsawa SF. Macrophage Polarization States in the Tumor Microenvironment. Int J Mol Sci (2021) 22(13):6995. doi: 10.3390/ijms22136995 34209703 PMC8268869

[62] Pan Y Yu Y Wang X Zhang T. Tumor-Associated Macrophages in Tumor Immunity. Front Immunol (2020) 11:583084. doi: 10.3389/fimmu.2020.583084 33365025 PMC7751482

[63] Ahirwar DK Charan M Mishra S Verma AK Shilo K Ramaswamy B. Slit2 Inhibits Breast Cancer Metastasis by Activating M1-Like Phagocytic and Antifibrotic Macrophages. Cancer Res (2021) 81(20):5255–67. doi: 10.1158/0008-5472.CAN-20-3909 PMC8631742 34400395

[64] Salmaninejad A Valilou SF Soltani A Ahmadi S Abarghan YJ Rosengren RJ. Tumor-Associated Macrophages: Role in Cancer Development and Therapeutic Implications. Cell Oncol (Dordr) (2019) 42(5):591–608. doi: 10.1007/s13402-019-00453-z 31144271 PMC12994359

[65] Shan K Feng N Cui J Wang S Qu H Fu G. Resolvin D1 and D2 Inhibit Tumour Growth and Inflammation *via* Modulating Macrophage Polarization. J Cell Mol Med (2020) 24(14):8045–56. doi: 10.1111/jcmm.15436 PMC7348143 32469149

[66] Zhang F Parayath NN Ene CI Stephan SB Koehne AL Coon ME. Genetic Programming of Macrophages to Perform Anti-Tumor Functions Using Targeted mRNA Nanocarriers. Nat Commun (2019) 10(1):3974. doi: 10.1038/s41467-019-11911-5 31481662 PMC6722139

[67] Bao X Shi R Zhao T Wang Y Anastasov N Rosemann M. Integrated Analysis of Single-Cell RNA-Seq and Bulk RNA-Seq Unravels Tumour Heterogeneity Plus M2-Like Tumour-Associated Macrophage Infiltration and Aggressiveness in TNBC. Cancer Immunol Immunother (2021) 70(1):189–202. doi: 10.1007/s00262-020-02669-7 32681241 PMC10992761

[68] Mehta AK Cheney EM Hartl CA Pantelidou C Oliwa M Castrillon JA. Targeting Immunosuppressive Macrophages Overcomes PARP Inhibitor Resistance in BRCA1-Associated Triple-Negative Breast Cancer. Nat Cancer (2021) 2(1):66–82. doi: 10.1038/s43018-020-00148-7 33738458 PMC7963404

[69] Liu C Chikina M Deshpande R Menk AV Wang T Tabib T. Treg Cells Promote the SREBP1-Dependent Metabolic Fitness of Tumor-Promoting Macrophages *via* Repression of CD8(+) T Cell-Derived Interferon-Gamma. Immunity (2019) 51(2):381–397.e6. doi: 10.1016/j.immuni.2019.06.017 31350177 PMC6703933

[70] Guo Q Jin Z Yuan Y Liu R Xu T Wei H. New Mechanisms of Tumor-Associated Macrophages on Promoting Tumor Progression: Recent Research Advances and Potential Targets for Tumor Immunotherapy. J Immunol Res (2016) 2016:9720912. doi: 10.1155/2016/9720912 27975071 PMC5128713

[71] Patarca R Freeman GJ Singh RP Wei FY Durfee T Blattner F. Structural and Functional Studies of the Early T Lymphocyte Activation 1 (Eta-1) Gene. Definition of a Novel T Cell-Dependent Response Associated With Genetic Resistance to Bacterial Infection. J Exp Med (1989) 170(1):145–61. doi: 10.1084/jem.170.1.145 PMC2189370 2787378

[72] Morimoto Y Hirahara K Kiuchi M Wada T Ichikawa T Kanno T. Amphiregulin-Producing Pathogenic Memory T Helper 2 Cells Instruct Eosinophils to Secrete Osteopontin and Facilitate Airway Fibrosis. Immunity (2018) 49(1):134–150.e6. doi: 10.1016/j.immuni.2018.04.023 29958800

[73] Kourepini E Aggelakopoulou M Alissafi T Paschalidis N Simoes DC Panoutsakopoulou V. Osteopontin Expression by CD103- Dendritic Cells Drives Intestinal Inflammation. Proc Natl Acad Sci U.S.A. (2014) 111(9):E856–65. doi: 10.1073/pnas.1316447111 PMC3948306 24550510

[74] Wang F Zhou Y Fu B Wu Y Zhang R Sun R. Molecular Signatures and Transcriptional Regulatory Networks of Human Immature Decidual NK and Mature Peripheral NK Cells. Eur J Immunol (2014) 44(9):2771–84. doi: 10.1002/eji.201344183 24838931

[75] Klement JD Paschall AV Redd PS Ibrahim ML Lu C Yang D. An Osteopontin/CD44 Immune Checkpoint Controls CD8+ T Cell Activation and Tumor Immune Evasion. J Clin Invest (2018) 128(12):5549–60. doi: 10.1172/JCI123360 PMC6264631 30395540

[76] Wei J Marisetty A Schrand B Gabrusiewicz K Hashimoto Y Ott M. Osteopontin Mediates Glioblastoma-Associated Macrophage Infiltration and is a Potential Therapeutic Target. J Clin Invest (2019) 129(1):137–49. doi: 10.1172/JCI121266 PMC6307970 30307407

[77] Klement JD Poschel DB Lu C Merting AD Yang D Redd PS. Osteopontin Blockade Immunotherapy Increases Cytotoxic T Lymphocyte Lytic Activity and Suppresses Colon Tumor Progression. Cancers (Basel) (2021) 13(5):1006. doi: 10.3390/cancers13051006 33670921 PMC7957528

[78] Sichien D Scott CL Martens L Vanderkerken M Van Gassen S Plantinga M. IRF8 Transcription Factor Controls Survival and Function of Terminally Differentiated Conventional and Plasmacytoid Dendritic Cells, Respectively. Immunity (2016) 45(3):626–40. doi: 10.1016/j.immuni.2016.08.013 27637148

[79] Waight JD Netherby C Hensen ML Miller A Hu Q Liu S. Myeloid-Derived Suppressor Cell Development is Regulated by a STAT/IRF-8 Axis. J Clin Invest (2013) 123(10):4464–78. doi: 10.1172/JCI68189 PMC3784535 24091328

[80] Qi J Sun H Zhang Y Wang Z Xun Z Li Z. Single-Cell and Spatial Analysis Reveal Interaction of FAP(+) Fibroblasts and SPP1(+) Macrophages in Colorectal Cancer. Nat Commun (2022) 13(1):1742. doi: 10.1038/s41467-022-29366-6 35365629 PMC8976074

[81] Chen P Zhao D Li J Liang X Li J Chang A. Symbiotic Macrophage-Glioma Cell Interactions Reveal Synthetic Lethality in PTEN-Null Glioma. Cancer Cell (2019) 35(6):868–884.e6. doi: 10.1016/j.ccell.2019.05.003 31185211 PMC6561349

[82] Rabe DC Walker ND Rustandy FD Wallace J Lee J Stott SL. Tumor Extracellular Vesicles Regulate Macrophage-Driven Metastasis Through CCL5. Cancers (Basel) (2021) 13(14):3459. doi: 10.3390/cancers13143459 34298673 PMC8303898

[83] Liang KH Yeh CT. OPN Sesame. Hepatobil Surg Nutr (2014) 3(3):112–4. doi: 10.3978/j.issn.2304-3881.2014.02.09 PMC4073313 25019071

[84] Zhu B Suzuki K Goldberg HA Rittling SR Denhardt DT McCulloch CA. Osteopontin Modulates CD44-Dependent Chemotaxis of Peritoneal Macrophages Through G-Protein-Coupled Receptors: Evidence of a Role for an Intracellular Form of Osteopontin. J Cell Physiol (2004) 198(1):155–67. doi: 10.1002/jcp.10394 14584055

[85] Feng F Rittling SR. Mammary Tumor Development in MMTV-C-Myc/MMTV-V-Ha-Ras Transgenic Mice is Unaffected by Osteopontin Deficiency. Breast Cancer Res Treat (2000) 63(1):71–9. doi: 10.1023/A:1006466516192 11079161

[86] Lin CN Wang CJ Chao YJ Lai MD Shan YS. The Significance of the Co-Existence of Osteopontin and Tumor-Associated Macrophages in Gastric Cancer Progression. BMC Cancer (2015) 15:128. doi: 10.1186/s12885-015-1114-3 25872762 PMC4384326

[87] Guilliams M De Kleer I Henri S Post S Vanhoutte L De Prijck S. Alveolar Macrophages Develop From Fetal Monocytes That Differentiate Into Long-Lived Cells in the First Week of Life *via* GM-CSF. J Exp Med (2013) 210(10):1977–92. doi: 10.1084/jem.20131199 PMC3782041 24043763

[88] Schulz C Gomez Perdiguero E Chorro L Szabo-Rogers H Cagnard N Kierdorf K. A Lineage of Myeloid Cells Independent of Myb and Hematopoietic Stem Cells. Science (2012) 336(6077):86–90. doi: 10.1126/science.1219179 22442384

[89] Hoeffel G Chen J Lavin Y Low D Almeida FF See P. C-Myb(+) Erythro-Myeloid Progenitor-Derived Fetal Monocytes Give Rise to Adult Tissue-Resident Macrophages. Immunity (2015) 42(4):665–78. doi: 10.1016/j.immuni.2015.03.011 PMC4545768 25902481

[90] Bian Z Gong Y Huang T Lee CZW Bian L Bai Z. Deciphering Human Macrophage Development at Single-Cell Resolution. Nature (2020) 582(7813):571–6. doi: 10.1038/s41586-020-2316-7 32499656

[91] Gomez Perdiguero E Klapproth K Schulz C Busch K Azzoni E Crozet L. Tissue-Resident Macrophages Originate From Yolk-Sac-Derived Erythro-Myeloid Progenitors. Nature (2015) 518(7540):547–51. doi: 10.1038/nature13989 PMC5997177 25470051

[92] Ginhoux F Greter M Leboeuf M Nandi S See P Gokhan S. Fate Mapping Analysis Reveals That Adult Microglia Derive From Primitive Macrophages. Science (2010) 330(6005):841–5. doi: 10.1126/science.1194637 PMC3719181 20966214

[93] Zhang Y Du W Chen Z Xiang C. Upregulation of PD-L1 by SPP1 Mediates Macrophage Polarization and Facilitates Immune Escape in Lung Adenocarcinoma. Exp Cell Res (2017) 359(2):449–57. doi: 10.1016/j.yexcr.2017.08.028 28830685

[94] Sharon Y Raz Y Cohen N Ben-Shmuel A Schwartz H Geiger T. Tumor-Derived Osteopontin Reprograms Normal Mammary Fibroblasts to Promote Inflammation and Tumor Growth in Breast Cancer. Cancer Res (2015) 75(6):963–73. doi: 10.1158/0008-5472.CAN-14-1990 25600648

[95] Butti R Nimma R Kundu G Bulbule A Kumar TVS Gunasekaran VP. Tumor-Derived Osteopontin Drives the Resident Fibroblast to Myofibroblast Differentiation Through Twist1 to Promote Breast Cancer Progression. Oncogene (2021) 40(11):2002–17. doi: 10.1038/s41388-021-01663-2 33603163

[96] Zhang L Li Z Skrzypczynska KM Fang Q Zhang W O'Brien SA. Single-Cell Analyses Inform Mechanisms of Myeloid-Targeted Therapies in Colon Cancer. Cell (2020) 181(2):442–459.e29. doi: 10.1016/j.cell.2020.03.048 32302573

[97] Buechler MB Fu W Turley SJ. Fibroblast-Macrophage Reciprocal Interactions in Health, Fibrosis, and Cancer. Immunity (2021) 54(5):903–15. doi: 10.1016/j.immuni.2021.04.021 33979587

[98] Duffield JS Forbes SJ Constandinou CM Clay S Partolina M Vuthoori S. Selective Depletion of Macrophages Reveals Distinct, Opposing Roles During Liver Injury and Repair. J Clin Invest (2005) 115(1):56–65. doi: 10.1172/JCI200522675 15630444 PMC539199

[99] Ma PF Gao CC Yi J Zhao JL Liang SQ Zhao Y. Cytotherapy With M1-Polarized Macrophages Ameliorates Liver Fibrosis by Modulating Immune Microenvironment in Mice. J Hepatol (2017) 67(4):770–9. doi: 10.1016/j.jhep.2017.05.022 28596109

[100] Vitale I Manic G Coussens LM Kroemer G Galluzzi L. Macrophages and Metabolism in the Tumor Microenvironment. Cell Metab (2019) 30(1):36–50. doi: 10.1016/j.cmet.2019.06.001 31269428

[101] Larionova I Kazakova E Gerashchenko T Kzhyshkowska J. New Angiogenic Regulators Produced by TAMs: Perspective for Targeting Tumor Angiogenesis. Cancers (Basel) (2021) 13(13):3253. doi: 10.3390/cancers13133253 34209679 PMC8268686

[102] Riabov V Gudima A Wang N Mickley A Orekhov A Kzhyshkowska J. Role of Tumor Associated Macrophages in Tumor Angiogenesis and Lymphangiogenesis. Front Physiol (2014) 5:75. doi: 10.3389/fphys.2014.00075 24634660 PMC3942647

[103] Conrad C Benzel J Dorzweiler K Cook L Schlomann U Zarbock A. ADAM8 in Invasive Cancers: Links to Tumor Progression, Metastasis, and Chemoresistance. Clin Sci (Lond) (2019) 133(1):83–99. doi: 10.BSR20182361/CS20180906 30635388

[104] Jaworek C Verel-Yilmaz Y Driesch S Ostgathe S Cook L Wagner S. Cohort Analysis of ADAM8 Expression in the PDAC Tumor Stroma. J Pers Med (2021) 11(2):113. doi: 10.3390/jpm11020113 33578644 PMC7916368

[105] Li Y Guo S Zhao K Conrad C Driescher C Rothbart V. ADAM8 Affects Glioblastoma Progression by Regulating Osteopontin-Mediated Angiogenesis. Biol Chem (2021) 402(2):195–206. doi: 10.1515/hsz-2020-0184 33544472

[106] Gjorgjevski M Hannen R Carl B Li Y Landmann E Buchholz M. Molecular Profiling of the Tumor Microenvironment in Glioblastoma Patients: Correlation of Microglia/Macrophage Polarization State With Metalloprotease Expression Profiles and Survival. Biosci Rep (2019) 39(6):BSR20182361. doi: 10.BSR20182361/BSR20182361 31142630 PMC6616040

[107] Rangaswami H Bulbule A Kundu GC. Osteopontin: Role in Cell Signaling and Cancer Progression. Trends Cell Biol (2006) 16(2):79–87. doi: 10.1016/j.tcb.2005.12.005 16406521

[108] Mirzaei A Mohammadi S Ghaffari SH Yaghmaie M Vaezi M Alimoghaddam K. Osteopontin B and C Splice Isoforms in Leukemias and Solid Tumors: Angiogenesis Alongside Chemoresistance. Asian Pac J Cancer Prev (2018) 19(3):615–23. doi: 10.22034/APJCP.2018.19.3.615 PMC5980831 29580029

[109] Ogata T Ueyama T Nomura T Asada S Tagawa M Nakamura T. Osteopontin is a Myosphere-Derived Secretory Molecule That Promotes Angiogenic Progenitor Cell Proliferation Through the Phosphoinositide 3-Kinase/Akt Pathway. Biochem Biophys Res Commun (2007) 359(2):341–7. doi: 10.1016/j.bbrc.2007.05.104 17537408

[110] Chakraborty G Jain S Kundu GC. Osteopontin Promotes Vascular Endothelial Growth Factor-Dependent Breast Tumor Growth and Angiogenesis *via* Autocrine and Paracrine Mechanisms. Cancer Res (2008) 68(1):152–61. doi: 10.1158/0008-5472.CAN-07-2126 18172307

[111] Takahashi F Akutagawa S Fukumoto H Tsukiyama S Ohe Y Takahashi K. Osteopontin Induces Angiogenesis of Murine Neuroblastoma Cells in Mice. Int J Cancer (2002) 98(5):707–12. doi: 10.1002/ijc.10261 11920639

[112] Dai J Li B Shi J Peng L Zhang D Qian W. A Humanized Anti-Osteopontin Antibody Inhibits Breast Cancer Growth and Metastasis *In Vivo* . Cancer Immunol Immunother (2010) 59(3):355–66. doi: 10.1007/s00262-009-0754-z PMC11030624 19690854

[113] Rittling SR Wejse PL Yagiz K Warot GA Hui T. Suppression of Tumour Growth by Orally Administered Osteopontin is Accompanied by Alterations in Tumour Blood Vessels. Br J Cancer (2014) 110(5):1269–77. doi: 10.1038/bjc.2014.10 PMC3950862 24473400

[114] Ishigamori R Komiya M Takasu S Mutoh M Imai T Takahashi M. Osteopontin Deficiency Suppresses Intestinal Tumor Development in Apc-Deficient Min Mice. Int J Mol Sci (2017) 18(5):1058. doi: 10.3390/ijms18051058 PMC5454970 28505114

[115] Anderson NR Minutolo NG Gill S Klichinsky M. Macrophage-Based Approaches for Cancer Immunotherapy. Cancer Res (2021) 81(5):1201–8. doi: 10.1158/0008-5472.CAN-20-2990 33203697

[116] Gao X Sheng Y Yang J Wang C Zhang R Zhu Y. Osteopontin Alters DNA Methylation Through Up-Regulating DNMT1 and Sensitizes CD133+/CD44+ Cancer Stem Cells to 5 Azacytidine in Hepatocellular Carcinoma. J Exp Clin Cancer Res (2018) 37(1):179. doi: 10.1186/s13046-018-0832-1 30064482 PMC6069805

[117] Bourassa B Monaghan S Rittling SR. Impaired Anti-Tumor Cytotoxicity of Macrophages From Osteopontin-Deficient Mice. Cell Immunol (2004) 227(1):1–11. doi: 10.1016/j.cellimm.2004.01.001 15051510

[118] Zhou KQ Liu WF Yang LX Sun YF Hu J Chen FY. Circulating Osteopontin Per Tumor Volume as a Prognostic Biomarker for Resectable Intrahepatic Cholangiocarcinoma. Hepatobil Surg Nutr (2019) 8(6):582–96. doi: 10.21037/hbsn.2019.03.14 PMC6943027 31929985

[119] Danzaki K Kanayama M Alcazar O Shinohara ML. Osteopontin has a Protective Role in Prostate Tumor Development in Mice. Eur J Immunol (2016) 46(11):2669–78. doi: 10.1002/eji.201646391 27601131

[120] Chan JM Quintanal-Villalonga A Gao VR Xie Y Allaj V Chaudhary O. Signatures of Plasticity, Metastasis, and Immunosuppression in an Atlas of Human Small Cell Lung Cancer. Cancer Cell (2021) 39(11):1479–1496.e18. doi: 10.1016/j.ccell.2021.09.008 34653364 PMC8628860

[121] Hansakon A Png CW Zhang Y Angkasekwinai P. Macrophage-Derived Osteopontin Influences the Amplification of Cryptococcus Neoformans-Promoting Type 2 Immune Response. J Immunol (2021) 207(8):2107–17. doi: 10.4049/jimmunol.2100202 34526375

